# Selection signature analyses and genome‐wide association reveal genomic hotspot regions that reflect differences between breeds of horse with contrasting risk of degenerative suspensory ligament desmitis

**DOI:** 10.1093/g3journal/jkac179

**Published:** 2022-07-22

**Authors:** Mehdi Momen, Sabrina H Brounts, Emily E Binversie, Susannah J Sample, Guilherme J M Rosa, Brian W Davis, Peter Muir

**Affiliations:** Department of Surgical Sciences, School of Veterinary Medicine, University of Wisconsin-Madison, Madison, WI 53706, USA; Department of Surgical Sciences, School of Veterinary Medicine, University of Wisconsin-Madison, Madison, WI 53706, USA; Department of Surgical Sciences, School of Veterinary Medicine, University of Wisconsin-Madison, Madison, WI 53706, USA; Department of Surgical Sciences, School of Veterinary Medicine, University of Wisconsin-Madison, Madison, WI 53706, USA; Department of Animal and Dairy Sciences, University of Wisconsin-Madison, Madison, WI 53706, USA; Department of Veterinary Integrative Biosciences, College of Veterinary Medicine and Biomedical Sciences, Texas A&M University, College Station, TX 77843, USA; Department of Surgical Sciences, School of Veterinary Medicine, University of Wisconsin-Madison, Madison, WI 53706, USA

**Keywords:** DSLD, selection signature, genome-wide categorical association, horse breeds, breed-assigned risk levels

## Abstract

Degenerative suspensory ligament desmitis is a progressive idiopathic condition that leads to scarring and rupture of suspensory ligament fibers in multiple limbs in horses. The prevalence of degenerative suspensory ligament desmitis is breed related. Risk is high in the Peruvian Horse, whereas pony and draft breeds have low breed risk. Degenerative suspensory ligament desmitis occurs in families of Peruvian Horses, but its genetic architecture has not been definitively determined. We investigated contrasts between breeds with differing risk of degenerative suspensory ligament desmitis and identified associated risk variants and candidate genes. We analyzed 670k single nucleotide polymorphisms from 10 breeds, each of which was assigned one of the four breed degenerative suspensory ligament desmitis risk categories: control (Belgian, Icelandic Horse, Shetland Pony, and Welsh Pony), low risk (Lusitano, Arabian), medium risk (Standardbred, Thoroughbred, Quarter Horse), and high risk (Peruvian Horse). Single nucleotide polymorphisms were used for genome-wide association and selection signature analysis using breed-assigned risk levels. We found that the Peruvian Horse is a population with low effective population size and our breed contrasts suggest that degenerative suspensory ligament desmitis is a polygenic disease. Variant frequency exhibited signatures of positive selection across degenerative suspensory ligament desmitis breed risk groups on chromosomes 7, 18, and 23. Our results suggest degenerative suspensory ligament desmitis breed risk is associated with disturbances to suspensory ligament homeostasis where matrix responses to mechanical loading are perturbed through disturbances to aging in tendon (*PIN1*), mechanotransduction (*KANK1*, *KANK2*, *JUNB*, *SEMA7A*), collagen synthesis (*COL4A1*, *COL5A2*, *COL5A3*, *COL6A5*), matrix responses to hypoxia (*PRDX2*), lipid metabolism (*LDLR*, *VLDLR*), and *BMP* signaling (*GREM2*). Our results do not suggest that suspensory ligament proteoglycan turnover is a primary factor in disease pathogenesis.

## Introduction

Degenerative suspensory ligament (SL) desmitis (DSLD) is an untreatable progressive idiopathic disorder that affects the connective tissue of the lower limbs in horses and often leads to euthanasia (Halper *et al.* 2006, 2011). DSLD is an important health and welfare problem that is of great concern to the community of owners of Peruvian Horses (PHs) (Peruvian Pasos) and DSLD-affected horses of other breeds. DSLD was first described in the PH, a highly predisposed breed, and has subsequently described in other breeds including the Arabian (AR), American Saddlebred, Quarter Horse (QH), Morgan, Thoroughbred (TB), Paso Fino, Akhal-Teke, and European Warmbloods (Mero and Pool 2002; Mero and Scarlett 2005). Prevalence of DSLD is breed-related; pony and draft breeds do not develop DSLD based on clinical knowledge and published reports; other athletic breeds, such as the AR and TB, have intermediate risk (Young 1993; Mero and Scarlett 2005; Halper *et al.* 2006, 2011; Luo *et al.* 2016). In certain PH bloodlines or families, DSLD prevalence is up to 40% (Luo *et al.* 2016).

**Fig. 1.**
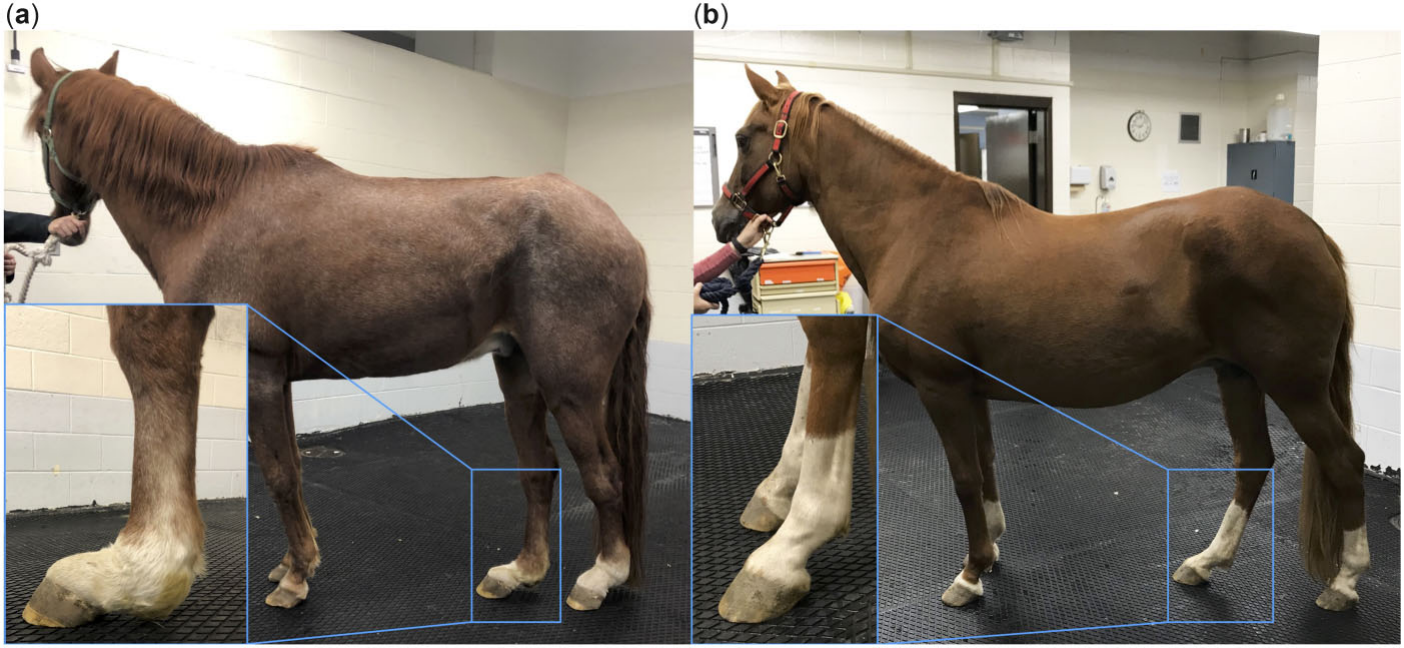
DSLD is a crippling, painful spontaneous equine disease. A Peruvian Horse (PH) (a) that is severely affected with DSLD and a (b) phenotype-negative control PH. As the disease develops, the SL progressively thickens. Over time, the SL mechanically weakens and ruptures, resulting in a classic sign of dropped fetlocks in multiple limbs. In the severe case, obvious thickening and dropping of the fetlocks is evident (inset a) compared with the normal standing posture (inset b). DSLD is typically more evident in the pelvic limbs verses the thoracic limbs, although in some PHs DSLD develops in all four limbs.

Onset of DSLD is subtle with progressive multilimb lameness developing with associated fetlock hyperextension and SL thickening (Halper *et al.* 2011). Age at diagnosis is variable, ranging from 3 to 17 years (Mero and Pool 2002). DSLD is characterized by increased diameter of the body and branches of the SL (Fig. 1). Collagen disruption, accumulation of interfibrillar matrix proteoglycans, and chondroid metaplasia are key histologic features in affected horses (Plaas *et al.* 2011). In the PH, DSLD can develop with no history of athletic work or SL injury. This suggests a specific etiology that includes a genetic contribution to disease risk given the strong breed predisposition in PHs (Mero and Scarlett 2005), although the genetic architecture of the disease is unclear.

Breed development of the horse has generated selection pressures to enable to work in agriculture and transport. Rare breeds can be exposed to loss of population size and genetic diversity (Ablondi *et al.* 2020). Morphological and performance traits have been targets for selective breeding (Petersen *et al.* 2013; de Simoni Gouveia *et al.* 2014). These genetic differentiation events have been generated by natural and artificial selection, shaping genomes individually over time with unique traits and specific genomic footprints. An unintended consequence of this selection is an increased incidence of disease within certain breeds. When comparing breeds with different disease risk, it is important to highlight the locus undergoing selection where deleterious allele frequency may be increasing, even if it has not yet reached fixation (Gurgul *et al.* 2019). Many spontaneous equine diseases, such as DSLD, closely mimic human heritable disorders. Tendon and ligament degeneration is an important health problem in humans and animals, but the contributing genes and pathways are poorly understood. Comparative studies of a disease in a spontaneous animal model where reduced genetic diversity within a breed is associated with long stretches of linkage disequilibrium (LD) can be advantageous as power of association is higher.

During past decades, investigation of the molecular pathology of DSLD, disturbances to signaling pathways, genome-wide analysis with low-density markers, and case–control gene expression analysis have been performed (Young *et al.* 2018; Mero and Pool 2002; Haythorn *et al.* 2020). A low-resolution genome-wide association study (GWAS) in the PH identified candidate loci on *Equus caballus* autosomes (ECAs) 6, 7, 11, 14, and 26 that did not meet genome-wide significance (Strong 2005; Metzger and Distl 2020). Therefore, improved understanding of the genetic contribution to DSLD is needed. Carrier horses can be inadvertently used for breeding before development of a late-onset disease condition, such as DSLD, causing a welfare concern because of substantial horse morbidity. Breed predisposition suggests that DSLD-associated genetic variants are enriched in the PH through linkage to desirable phenotypes. From an evolutionary perspective, selection for a desired phenotype through breeding practices results in an increased frequency of haplotypes containing the gene(s) and functional allele(s) conferring the phenotype at a rate higher than expected under a null model of neutral evolution (Cutter and Payseur 2013). GWAS and detection of signatures of selection (SOS) are two complimentary approaches for association between a disease phenotype and genetic variation (Patron *et al.* 2019). In a comparative analysis, we investigated the breed risk of DSLD using a categorical genome-wide SOS and GWAS approach in the PH and nine other breeds using breed-assigned risk levels that reflect varying levels of breed risk of DSLD, to capitalize on the availability of public single-nucleotide polymorphism (SNP) data from different breeds of horse. A candidate locus from GWAS that localizes in a region with a signature of positive selection signal indicates potential influential variation, particularly for simple Mendelian diseases (Kemper *et al.* 2014).

DSLD heritability has not been estimated and no associated genetic markers have been identified. Genetic testing for DSLD is not available. Clinically, it is recommended not to use affected horses for breeding. Our rationale for this work was that large effect DSLD genetic variants that have become highly frequent in susceptible breeds should exhibit association signals in a categorical across breed study examining horses classified using average phenotypes for varying breed related DSLD risk. A locus under natural or artificial selection would be expected to contain influential variation. To test this hypothesis, we quantified the rate and strength of positive SOS and GWAS signals for breed-related risk of DSLD in 10 horse breeds classified by levels of risk. The work is innovative because as it departs from the usual case–control GWAS approach by performing across breed categorical genome-wide association using breed-assigned risk levels for breed risk of DSLD. We identified novel candidate genes for breed risk of DSLD that are involved in disturbances to aging in tendon, mechanotransduction, collagen synthesis, matrix responses to hypoxia, and lipid metabolism. Such genes are targets for investigation in human tendon/ligament degeneration and could form the basis for polygenic risk score prediction of disease risk.

## Materials and methods

### Sampling

Client-owned PHs were recruited at the UW Madison School of Veterinary Medicine, Texas A&M College of Veterinary Medicine, and through online advertising. Pedigrees were used to confirm purebred status. Blood samples or hair bulb samples pulled from the tail or mane were collected from 162 PHs. All owners gave informed consent to participate in the study. Buffy coat or hair bulbs underwent DNA isolation using the Gentra Puregene reagents (Qiagen, Valencia, CA). Concentrated DNA was stored at −20°C for genotyping. SNP genotyping was performed using the Axiom Equine Genotyping Array (Axiom MNEC670K), which includes a total of 670,796 SNPs across all chromosomes. SNP genomic coordinates were based on the latest genome assembly, EquCab3 (Kalbfleisch *et al.* 2018). Two additional datasets, described below, were also used by obtaining genotypes for the relevant SNP positions from published whole-genome sequence and high-density genotype data.

Genotypic data were obtained from 304 horses representing nine breeds including: TB (*n* = 28), QH (*n* = 72), Belgian (BE) (*n* = 22), AR (*n* = 36), Welsh Pony (WP) (*n* = 44), Standardbred (SB) (*n* = 39), Icelandic Horse (IH) (*n* = 18), Lusitano (LU) (*n* = 21), and Shetland Pony (SP) (*n* = 24) (Table 1). For all breeds other than the SP, data were previously generated during development of the high-density equine SNP array (Schaefer *et al.* 2017). In this study, whole-genome sequence data compiled from 153 horses representing 24 separate breeds were used to discover ∼23 million biallelic candidate SNPs. After quality control and filtration based on breed representation, even spacing across the genome, and probe design considerations, 2,001,826 SNPs were selected for the Affymetrix equine MNEc2M SNP array (Schaefer *et al.* 2017). Of these SNPs, we selected those SNPs shared with Axiom MNEc670 that were located on ECAs 1–31. Horse samples from 24 SPs provided by Dr. Rebecca R. Bellone, from Veterinary Genetics Laboratory, School of Veterinary Medicine, University of California, Davis were also included in this study. These samples were genotyped using the Axiom Equine Genotyping 670K array and were remapped to EquCab3 reference genome (Tanaka *et al.* 2019).

**Table 1.** Breeds of horse used for signature of selection (SOS) and genome-wide association study (GWAS) for equine DSLD after disease risk was assigned to each breed based on clinical knowledge.

Breed	# horses	Source	Horse type	DSLD risk	Categorical risk score
Arabian	36	Beeson *et al.* (2019) and Cosgrove *et al.* (2020)	Ancient	Low	1
Belgian	22	Beeson *et al.* (2019)	Draft	Control	0
Icelandic Horse	18	Beeson *et al.* (2019)	Pony	Control	0
Lusitano	21	Beeson *et al.* (2019)	Ancient	Low	1
Peruvian Horse	162	University of Wisconsin-Madison, Comparative Genetics & Orthopaedic Research Laboratory	Ambling	High	3
Quarter Horse	72	Beeson *et al.* (2019)	Athletic	Medium	2
Shetland Pony	24	Tanaka *et al.* (2019)	Pony	Control	0
Standardbred	39	Beeson *et al.* (2019)	Athletic	Medium	2
Thoroughbred	28	Beeson *et al.* (2019)	Athletic	Medium	2
Welsh Pony	44	Beeson *et al.* (2019)	Pony	Control	0

### Genetic population structure

We investigated patterns of population structure using five methods.

#### Principal component analysis

The genomic information was used to compute a genotypic (co)variance matrix between all individuals (Vitezica *et al.* 2013). By performing eigen decomposition on the matrix using the base eigen() function in R (R Core Team 2021), the eigenvectors and eigen values were obtained and normalized dividing each component of the vector by the length of the vector to vectors with a length of 1. Finally, PCs were computed by multiplying eigenvectors by the square root of the associated eigenvalues (Scholkopf *et al.* 1997). To review the results, we plotted the projection of the individuals on the first two PCs, with colors corresponding to their breed assignment.

#### Phylogenetic tree analysis

A pairwise identity-by-state distance matrix was computed using PLINK v1.09 and the –genome command followed by the –cluster command (Chang *et al.* 2015). To produce a bootstrapping procedure, we resampled 500 datasets with replacement from the original genotypes. Neighbor-joining cladograms were constructed with these matrices using PHYLIP (Felsenstein 1993). The “consense” program in PHYLIP was used to combine the bootstrap results and build a majority rule consensus tree. Cladograms were built using iTOL v6 RRID: SCR_018174 (Letunic and Bork 2021).

#### Admixture analysis

A maximum-likelihood based approach, was used to infer the ancestry proportions population using ADMIXTURE RRID: SCR_001263 (Alexander *et al.* 2009). To identify the most likely number of ancestral populations (*K*) in the dataset, a series of runs with predefined *K* values ranging from *K* = 3 to 30, were examined using 20 cross-validation runs (CV = 20). The termination convergence criterion (delta) was set to $10^{-4}$ to stop when the log-likelihood increases by less than this value between two consequent iterations.

#### Effective population size

Effective population size (Ne) trajectories were estimated from LD, across the horse breeds. We used SneP v1.1 (Barbato *et al.* 2015) to estimate breed-specific Ne trajectory, as an indicator of genetic drift and population demography in recent years for each population. The software estimates the historic effective population size based on the relationship between LD ($r^{2})$, Ne, and recombination rate (Sved 1971; Weir and Hill 1980).

#### LD-decay estimation

To measure LD content across breeds, we computed pairwise LD based on SNP $r^{2}$ using a window size of 100kb, using all markers with a minor allele frequency (MAF) ≥5% on each chromosome using PLINK v1.9 (Chang *et al.* 2015). Then, we fitted a nonlinear spline regression (Van Inghelandt *et al.* 2011; Vos *et al.* 2017). All chromosomes were concatenated to get an overall genome-wide LD-decay estimation.

We also generated a genomic relationship matrix (GRM) for each breed of horse to evaluate within-breed subpopulation structure (R Core Team 2021) using the method of VanRaden (2008).

### DSLD risk categorization

Contrasts between breed risk groups were used to identify DSLD-associated regions across the genome. We classified each breed into one of the four DSLD risk categories which were then used for individual horses belonging to that breed: control (1) (BE, IH, SP, and WP), low risk (2) (LU, AR), medium risk (3) (SB, TB, QH), and high risk (4) (PH). These breed risk phenotypes were then used for ordinal GWAS. This risk coding reflects current clinical knowledge of DSLD, of which the authors have extensive experience, where draft horses and pony breeds are protected from DSLD, certain athletic breeds have intermediate risk, and other breeds have high risk (Young 1993; Mero and Scarlett 2005; Halper *et al.* 2006, 2011; Luo *et al.* 2016). Some of the breeds in the public SNP dataset were discarded if clinical and epidemiological knowledge could not estimate DSLD risk for that breed.

### Selection and differentiation analyses

Since we were interested in selection along the evolutionary branch leading to breeds with high risk of DSLD, the control population was considered as the reference for comparison. As many approaches have been suggested, we scanned the genome for multiple patterns of molecular variation by (1) locally elevated levels of genetic differentiation based on allele frequency differences and (2) differences in long-range haplotype frequencies between risk groups. To infer these types of signatures, we estimated levels and patterns of genetic diversity and differentiation using two approaches. A pairwise population haplotype frequency test using hapFLK (Fariello *et al.* 2013), which accounts for the hierarchical structure of the sampled populations, was used. Sex chromosomes were excluded from phasing, so haplotype statistics were limited to autosomes. We also used a window-based fixation index (*F*_ST_) that is a measurement of population differentiation due to genetic structure (Weir and Cockerham 1984). Finally, we generated locus-specific diagrams of SOS positive hotspot regions reflecting LD as well as position relative to nearby tendinopathy candidate genes.

### hapFLK

We performed hapFLK tests to contrast the high risk group with each of the control, low risk and medium risk groups using hapFLK v1.2 (Fariello *et al.* 2013). Computation of hapFLK proceeds in three steps. First, we estimated a kinship matrix across groups, which was calculated using the Reynold’s genetic distances approach. For each SNP in the genome and across risk groups, we then performed the hapFLK test that incorporates haplotypic information to increase the power to detect selective sweeps, where a new mutation that increases its frequency and becomes quickly fixed in the population such that linked alleles also become fixed. So, the hapFLK statistic calculates the deviation of haplotypic frequencies with respect to the neutral model estimated by the kinship matrix (Reynolds *et al.* 1983). To exploit LD information, hapFLK uses a multipoint model for multilocus genotypes that can be fitted to unphased data (Scheet and Stephens 2006). One of the main applications of this model is to perform phase estimation using fastPHASE (Scheet and Stephens 2006). In our analysis, the model was trained on unphased data and, therefore, our analysis accounts for phase uncertainty associated with estimation of LD content during haplotype analysis.

The method was used to regroup local haplotypes along chromosomes in a specified number of clusters K set to 25, using a Hidden Markov Model. We used the following parameters: 25 clusters (-K 25), 20 EM (expectation maximization) runs to fit the LD model (-nfit = 20). Once hapFLK values were generated we implement the “rglm()” function from MASS R package to fit a robust linear model to normalize hapFLK scores and calculate *P*-values. hapFLK *P*-values at each SNP were computed from this estimated distribution. To identify region of interest, we used a Bonferroni-corrected threshold of *P *≤* *1.13E−07, obtained by dividing *P < *0.05 by the number of SNPs in the model.

### Fixation index (*F*_ST_)

The fixation index (Weir and Cockerham 1984) is a moment estimator of *F*_ST_ and uses unbiased estimators of the numerator (between-population variance) and the denominator (total variance). Here, we used the *F*_ST_ statistic to identify unique, divergent regions of the affected population showing differentiation across risk groups. *F*_ST_ was calculated in sliding windows of 50kb and a step size of 25kb using VCFtools 0.1.15 (Danecek *et al.* 2011). Then, the estimated *F*_ST_ values were scaled in R software to follow a normal distribution. Standard score or *z*-score is a measure of standard deviation that estimates the distance from the mean. $Z\left(F_{\mathrm{ST}}\right)=\frac{F_{\mathrm{ST}\left(i\right)}-\mu_{{(F}_{\mathrm{ST}})}}{\mathrm{SD}(F_{\mathrm{ST}})}$, here, μ is mean value of all computed *F*_ST_ and SD(*F*_ST_) is the standard deviation of them. Then, we defined a stringent cut off value and selected only the 0.005 most divergent windows.

### Liability threshold modeling of ordinal DSLD status for across breed GWAS

We also employed a liability categorical threshold (cumulative probit) model. The response variable (disease risk category), $y_{ik}$, represents an assignment into one of the four mutually exclusive and exhaustive categories that follow an order, where 1 indicates no risk (control), 2 low risk, 3 moderate risk, and 4 high risk. A schematic representation of this model is shown in Supplementary Fig. 1. Therefore, in a GLMM framework, this model can be described by defining the distribution, the linear predictor and a link function. This threshold model assumes that the process that gives rise to the observed categories is an underlying continuous variable with a normal distribution:

$$l_{\mathrm{ijk}}=x_{ij}^{T}\beta +z_{ij}^{T}b+\varepsilon_{\mathrm{ijk}}$$

where $\;l_{\mathrm{ijk}}$ represents liabilities, conditionally independent and distributed as$\;l_{\mathrm{ijk}}|\beta ,b∼N(x_{ij}^{T}\beta +z_{ij}^{T}b,\;\sigma_{\varepsilon }^{2}=1)$, *x_ij_* denotes the *j*-th row of the fixed effects design matrix *X_i_*, with the corresponding fixed effects coefficients denoted by ***β***, *z_ij_* denotes the *j*-th row of the random effects design matrix *Z_i_* with corresponding random effects ***b***, and $\varepsilon_{\mathrm{ijk}}$ is the error term, which follows a normal distribution as $\varepsilon_{\mathrm{ijk}}∼N(0,1)$; $\sigma_{\varepsilon }^{2}\;$was set to 1 to achieve variance identifiability in the likelihood because the liabilities are unobservable (Gianola 1982; Sorensen *et al.* 1995). Also, the b vector was assumed to follow the normal distribution $b∼N(0,G\sigma_{g}^{2})$, where ***G*** is a marker-derived GRM, and $\sigma_{g}^{2}$, represents genomic variance. The ordinal categorical phenotypes were mapped to four categories (*C* = 4), based on the threshold parameters $\gamma^{T}=\left(\gamma_{\min}<\gamma_{1}<\ldots <\gamma_{C-1}<\gamma_{\max}\right)$, with $\gamma_{\min}=-\infty$ and $\gamma_{\max}=+\infty$, which are cut points of the continuous scale such that the assigned ordinal disease risk category ($y_{ik}$) is given by:

$$y_{ik}=\;\left\{\begin{array}{cccccc} 1 & if & -\infty < & l_{\mathrm{ijk}} & \leq & \gamma_{1}\\ 2 & if & \gamma_{1}< & l_{\mathrm{ijk}} & \leq & \gamma_{2}\\ 3 & if & \gamma_{2}< & l_{\mathrm{ijk}} & \leq & \gamma_{3}\\ 4 & if & \gamma_{3}< & l_{\mathrm{ijk}} & < & \infty \end{array}\right.$$

This model was implemented via a Bayesian approach using the Gibbs sampler algorithm, and sampling from the fully conditional distributions (Sorensen *et al.* 1995) in the BGLR R package (Perez and de Los Campos 2014; R Core Team 2021).

We then implemented a univariate linear mixed model regression GWAS model using the “gaston” R package (Dandine-Roulland and Perdry 2017) to test each single genetic variant independently. We regressed each SNP and used the Wald test to determine *P*-values. We included a GRM using the “GRM()” internal function from the “gaston” package to correct for population structure and cryptic relatedness and remove potential sources of spurious associations. After obtaining the *P*-values, we computed the chi-squared statistic (chisq<-qchisq(1-data$pval,1) and then the genomic inflation factor (λ) (median of the resulting chi-squared test statistics divided by the expected median of the chi-squared distribution with one degree of freedom (df = 1) (median(chisq)/qchisq(0.5,1)) using R (R Core Team 2021) to investigate possible systematic bias in our association results and reviewed results using a quantile–quantile (*Q–Q*) plot.

Next, we considered two approaches to define significant thresholds. First, a Bonferroni correction was used (*P *≤* *1.13E−07). However, Bonferroni correction may be too conservative as each SNP is not an independent test when SNPs reside within long blocks of LD. As an alternative, we defined genome-wide significance using 95% confidence intervals (CI) calculated from the empirical distribution of *P*-values observed in the absence of real association (Karlsson *et al.* 2013). We determined this distribution by rerunning the GWAS with randomly permuted phenotypes 500 times. We defined genome-wide significance as associations exceeding the 5% upper empirical CI (*P *≤* *3.24E−5).

Finally, we recalculated principal component analysis (PCA) as described above using the GWAS SNPs with *P*-values lower than the permutation threshold for association with breed risk of DSLD and plotted the projection of the horses on the first two PCs, with colors corresponding to their breed assignment.

### Candidate gene identification and pathway analysis

The biological functions of genes mapped to the human genome and located within positive SOS genomic regions or within flanking regions (50kb) of GWAS-associated SNPs were screened for relevance to tendon/ligament homeostasis. We also used the University of California Santa Cruz genome browser (https://genome.ucsc.edu/) to map the significant SNPs that were found to be under positive selection and associated with breed group risk of DSLD to the EquCab3.0 assembly (Casper *et al.* 2018). Lists of associated genes were submitted for pathway analysis using Panther software (Mi *et al.* 2013, 2019).

## Results

### Genetic diversity and population structure

**Fig. 2.**
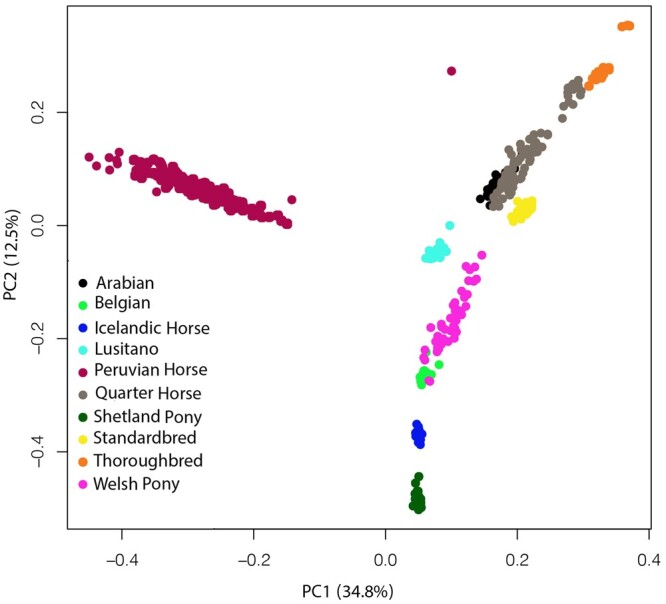
A Principal component analysis (PCA) plot representing the genetic landscape of 10 horse breeds extended across first and second components (PC1 and PC2) derived from eigen vectors and eigen values obtained from eigen decomposition of a genotypic (co)variance matrix between all individuals. Each color shows a different breed, and each point represents 1 sample.

The population architecture and substructures were investigated using several approaches as a prerequisite for SOS analysis. Genomic relationship matrices were plotted for each breed to evaluate population substructure (Supplementary Fig. 2). PCA showed good differentiation of the 10 horse breeds by the first and second principal components (PC1, PC2) (Fig. 2). The first two PCs explained 34.8% and 12.5% of total genetic variation, respectively. Adding additional PCs did not result in any further observable genetic clusters (Supplementary Fig. 3). The Peruvian Horse breed was separated from other breeds using PC1. The SP, IH, BE, WP, and LU breeds were separated along PC2. The PC results showed a great overlap between AR and QH breeds, indicating a close genetic relationship between these two breeds in our data. Furthermore, SB, QH, AR, and TB breeds showed a closer relationship with each other than with other breeds along both PC1 and PC2.

**Fig. 3.**
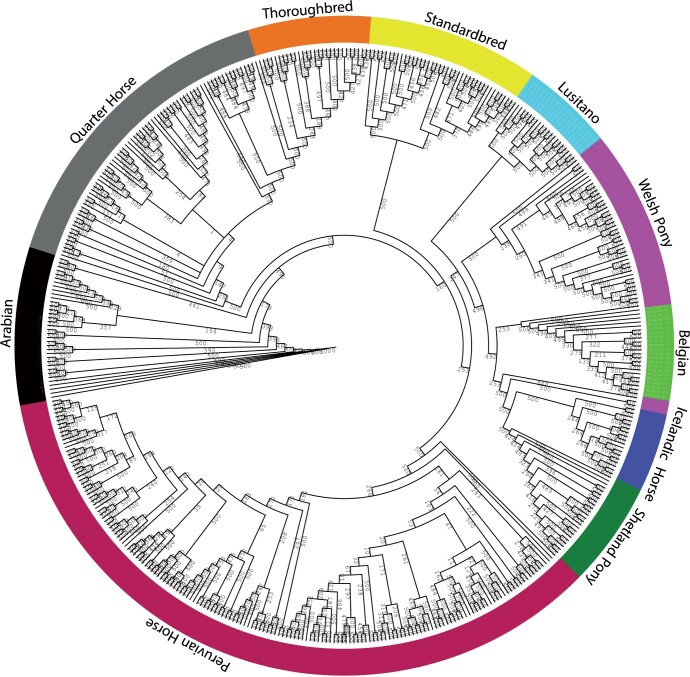
Cladogram of 10 horse breeds obtained using a bootstrapped procedure by identity-by-state distance matrix (IBS) and a neighbor-joining tree algorithm. The outer circle color indicates how breeds constituted their own branches. The number on the branches and sub-branches indicates how they were supported by 500 bootstrap replicates.

We also assessed population history for admixture events using model-based ancestry clustering. The ADMIXTURE software was used to determine the number of genetic background ancestors (*K*) that explain the observed total sum of between-breed genetic variation. For SOS analysis, it is important to know the composition of breed ancestry. To estimate the optimum number of ancestors, we tested a range of *K* values from 3 to 33. The lowest 20-fold cross-validation mean squared error (MSE) was obtained at *K *=* *14 (MSE = 0.431, Supplementary Fig. 4). There were only minor differences between the four *K* values, *K *=* *8, 12, 14, and 18 with the lowest MSE (Supplementary Fig. 4); results from *K *=* *12, 14, and 18 had less than 0.001 MSE differences. This analysis indicated the presence of at least 12 genetic backgrounds. The individual assignment probabilities indicate distinct genetic backgrounds exist for the AR, SB, TB, BE, and IH, suggesting clear genetic divergence. This was evident across the four lowest values of *K* tested. In all these scenarios, the SB, PH, LU, and AR showed some degree of ancestral gene flow based on admixture analysis (Supplementary Fig. 4). For the PH, our PCA results show that ancestors are distant and different from the other breeds in the data set, different from the admixture analysis. All 10 breeds formed single, breed-specific nodes with good bootstrap support (Figs. 2 and 3). Like the PCA plot, the identity by descent similarity revealed a close genetic relationship between AR, QH, TB, SB, WP, SP, LU, BE, and IH breeds. Also, the PH resides in a distinct clade. We observed horses from the same breed almost perfectly clustered together, but slight differences were found in the internal branches within a breed. Next, we estimated patterns of LD decay among clusters over all 31 autosomal chromosomes and found the average correlation coefficient (*r*^2^), generally decayed rapidly. The decay trend differed between breeds (Supplementary Fig. 5). The largest LD was observed in the TB and the lowest in the PH. The breeds with second and third largest LD content were the SP and AR, respectively. Other breeds with the smallest LD were the WP and the QH.

To further investigate differences in LD content across breeds, we estimated ancestral and recent effective population sizes (Ne), which is an important genetic parameter that relates to the amount of LD content, genetic variation and genetic drift in a population and represents the minimum number of breeding individuals in an idealized population. The estimated effective population sizes for breeds over the past 100 generations showed an increasing trend across generations for effective population size and the Ne parameter diversified between breeds (Supplementary Fig. 6). The QH, PH, and WP breeds had larger Ne values, respectively, although the PH had a higher slope, than other breeds and after generation 75 had the highest Ne estimates. The lowest Ne estimates were obtained for TB, SP, and SB breeds. The Ne estimates agreed with LD content among breeds.

### Candidate regions and genes under positive selection in horse breeds with differing breed risk of DSLD

**Fig. 4.**
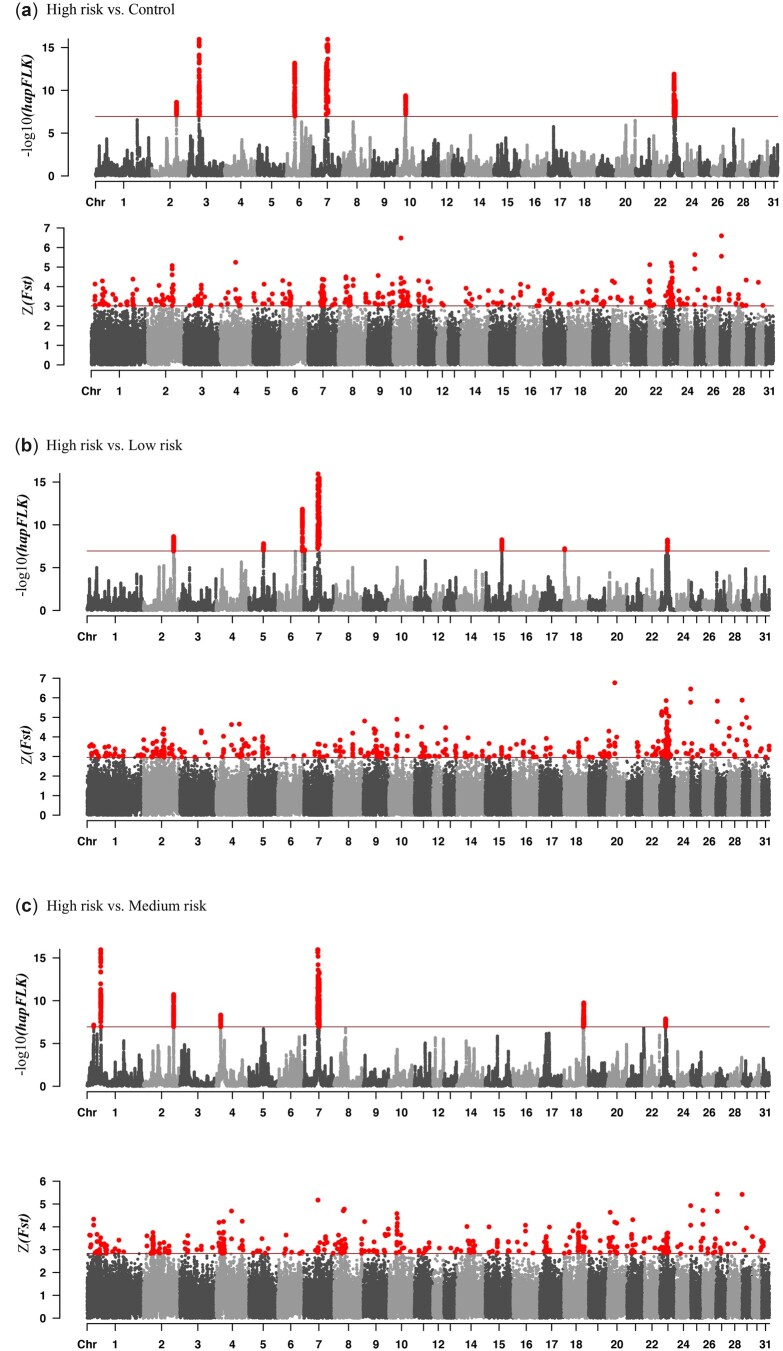
Manhattan plot of hapFLK and *F*_ST_ analysis over the 31 autosomal chromosomes across 3 group comparisons for the high risk (Peruvian Horse), medium risk (Standardbred, Thoroughbred, Quarter Horse), low risk (Lusitano, Arabian), and control (Belgian, Icelandic Horse, Shetland Pony, Welsh Pony) breed groups. a) High risk vs. control, b) high risk vs. low risk, and c) high risk vs. medium risk. The red line in the hapFLK Manhattan plot indicates the Bonferroni threshold line and in *F*_ST_ shows the upper 0.005% of the top windows of *F*_ST_ values distribution. The *x*-axes show the chromosomes and the *y*-axis the –log10(*P*-value) for hapFLK and *Z*-score based for *F*_ST_. All SNPs and windows passed the defined thresholds highlighted with red color.

**Fig. 5.**
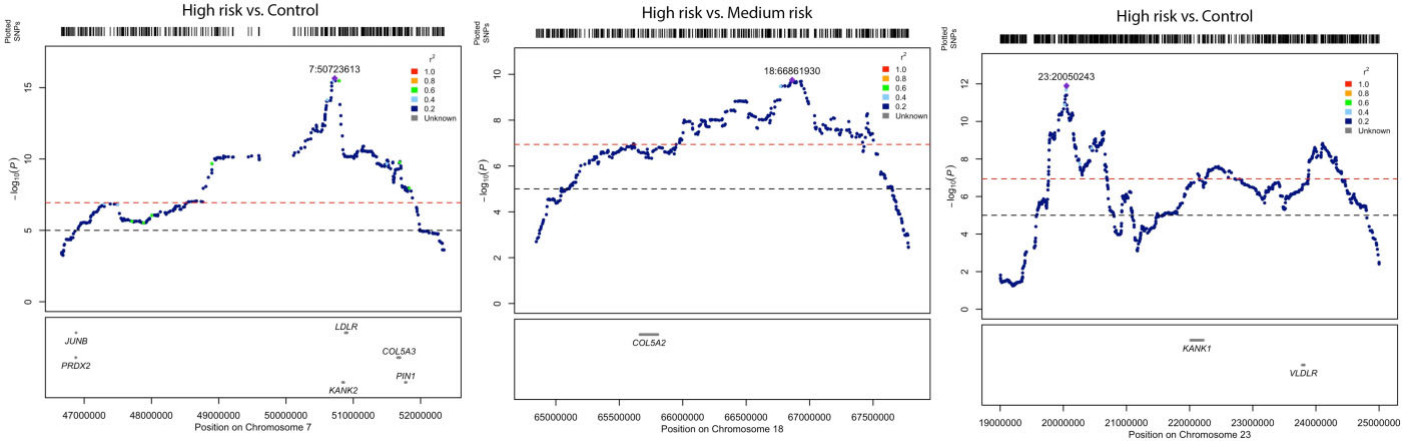
Locus Zoom plot of hotspot regions containing eight tendinopathy-related candidate genes for breed group risk of degenerative suspensory ligament desmitis on ECA7, 18, and 23. Each point represents an SNP. The color of each SNP indicates its LD quality, as indicated by color index tab. The most significant SNP in each region is indicated by a purple diamond.

The hapFLK analysis identified 6 regions under positive selection, which passed the Bonferroni-corrected threshold when the breed group with high risk of DSLD was compared with the control breed group (i.e. PH vs. BE, IH, SP, and WP) and distributed on ECA2, 3, 6, 7, 10, and 23 (Fig. 4). These regions were also detected when the breed group with high risk of DSLD was compared with low and medium risk breed groups. The largest regions were located on the ECA7 and 23, with a length of ∼3.3 and ∼4.6Mb, respectively (Table 2). The region on ECA7 contained the *COL5A3* (collagen type V alpha 3 chain), *KANK2* (KN Motif and Ankyrin Repeat Domains 2), *LDLR* (low-density lipoprotein receptor), and *PIN1* (Peptidyl-prolyl cis-trans isomerase NIMA-interacting 1) genes (Fig. 5).

**Table 2.** Significant signature of selection regions identified using hapFLK, across four DSLD risk groups, high risk vs. control (HR-Cont), high risk vs. low risk (HR-LR), and high risk vs. medium risk (HR-MR).

	Chr	Window size (bp)	# SNPs in window	*P*-Value	Genes
HR-Cont	2	82,375,201–82,484,566	36	2.481E−09	SH3D19
	3	36,714,694–37,237,743	38	1.110E−16	CHMP1A/DBNDD1/DEF8/FANCA/VPS9D1/TUBB3/TCF25/SPIRE2/CPNE7/PRDM7/MC1R/SPATA33/CENPBD1/CDK10/DPEP1/SPATA2L/GAS8/ZNF276
	6	28,042,417–29,187,416	145	6.505E−14	ATP6V1E1/CECR2/USP18/SLC25A18/MICAL3/TMEM121B/ADA2/CACNA1C/HDHD5/PEX26/BCL2L13/IL17RA/BID/TUBA8
	7^a^	48,550,747–51,878,056	197	1.110E−16	**PIN1**/CCDC151/ANGPTL6/ECSIT/C7H19orf66/UBL5/ZGLP1/CARM1/ZNF653/CCDC159/RAVER1/S1PR2/SPC24/TMED1/ATG4D/TYK2/CDKN2D/EPOR/ELAVL3/ACP5/SMARCA4/PRKCSH/YIPF2/CNN1/ICAM1/DNM2/SLC44A2/CDC37/EIF3G/**LDLR**/C7H19orf38/ICAM3/PLPPR2/ILF3/MRPL4/FDX1L/**KANK2**/RDH8/ICAM4/KEAP1/TMEM205/TIMM29/**COL5A3**/FBXL12/DNMT1/ICAM5/DOCK6/P2RY11/ELOF1/RAB3D/OLFM2/TSPAN16/RGL3/QTRT1/AP1M2/SWSAP1/PDE4A/KRI1/S1PR5/ANGPTL8
	10	27,798,713–28,288,061	58	4.195E−10	ZNF135/ZNF274/ZNF606/C10H19orf18/ZNF671
	23^a^	19,766,482–24,404,305	257	1.261E−12	PTAR1/FXN/TMEM252/SMARCA2/PUM3/**VLDLR**/**KANK1**/PIP5K1B/DMRT3/FAM189A2/DOCK8/TJP2/FAM122A/DMRT1/C23H9orf135/MAMDC2/KCNV2/DMRT2/RFX3/PGM5/KLF9/APBA1/SMC5
HR-LR	2	100,908,612–101,332,274	120	2.322E−09	PGRMC2/LARP1B/ABHD18
	5	49,161,652–49,553,142	58	1.557E−08	ATP1A1/MAB21L3/SLC22A15
	6	82,388,851–82,631,911	78	1.470E−12	HMGA2/MIR763
	7^a^	47,027,389–51,858,393	190	1.110E−16	**PIN1**/CCDC151/ANGPTL6/ECSIT/C7H19orf66/UBL5/ZGLP1/CARM1/ZNF653/CCDC159/RAVER1/S1PR2/SPC24/TMED1/ATG4D/TYK2/CDKN2D/EPOR/ELAVL3/ACP5/SMARCA4/PRKCSH/YIPF2/CNN1/ICAM1/DNM2/SLC44A2/CDC37/EIF3G/**LDLR**/C7H19orf38/ICAM3/PLPPR2/ILF3/MRPL4/FDX1L/**KANK2**/RDH8/ICAM4/KEAP1/TMEM205/TIMM29/**COL5A3**/FBXL12/DNMT1/ICAM5/DOCK6/P2RY11/ELOF1/RAB3D/OLFM2/TSPAN16/RGL3/QTRT1/AP1M2/SWSAP1/PDE4A/KRI1/S1PR5/ANGPTL8
	15	55,722,740–55,823,249	24	5.410E−09	LRPPRC/ABCG8/ABCG5/DYNC2LI1
	18	3,198,948–3,226,216	11	6.027E−08	MYO7B
	23^a^	26,575,733–26,190,830	36	5.861E−09	PDCD1LG2/RIC1/ERMP1
HR-MR	1	22,168,393–22,174,156	3	6.895E−08	None
	1	45,343,494–46,187,762	145	1.110E−16	PCDH15
	2	100,839,946–101,131,385	99	1.869E−11	PGRMC2
	4	15,433,715–15,886,714	52	4.632E−09	PURB/MYO1G/CCM2/NACAD/TBRG4/RAMP3
	7^a^	46,267,067–51,832,122	306	1.110E−16	**PIN1**/MAN2B1/CACNA1A/CCDC151/NACC1/RAD23A/ECSIT/STX10/ZGLP1/CCDC159/RAVER1/TRIR/S1PR2/**JUNB**/CDKN2D/DNASE2/ACP5/SMARCA4/PRKCSH/RNASEH2A/CNN1/DNM2/SLC44A2/IER2/KLF1/EIF3G/C7H19orf38/ICAM3/PLPPR2/MRPL4/**KANK2**/HOOK2/ICAM4/TMEM205/**COL5A3**/ASNA1/DAND5/FBXL12/DNMT1/ICAM5/P2RY11/LYL1/TSPAN16/NFIX/QTRT1/PDE4A/WDR83/ANGPTL6/C7H19orf66/UBL5/ZNF653/CARM1/SPC24/GADD45GIP1/BEST2/TMED1/MAST1/ATG4D/TYK2/GCDH/GNG14/EPOR/TRMT1/DHPS/ELAVL3/FBXW9/RTBDN/YIPF2/ICAM1/WDR83OS/CDC37/FARSA/**LDLR**/SYCE2/ILF3/FDX1L/TNPO2/RDH8/KEAP1/TIMM29/**PRDX2**/DOCK6/CALR/ELOF1/CCDC130/RAB3D/OLFM2/RGL3/AP1M2/SWSAP1/KRI1/S1PR5/ANGPTL8
	18	65,611,515–67,501,715	192	1.775E−10	WDR75/ORMDL1/STAT4/GLS/NAB1/SLC40A1/MFSD6/**COL5A2**/STAT1/MSTN/INPP1/ASNSD1/PMS1/C18H2orf88/HIBCH/ANKAR/OSGEPL1/NEMP2
	23	19,864,050–20,127,983	37	1.298E−08	SMC5/MAMDC2

a Regions shared between risk groups. Genes highlighted in bold underlined text may have biological effects on tendon and ligament homeostasis.

The second largest region seen in the high risk vs. control breed group comparison, located on the ECA23, contained the *KANK1* (KN motif and ankyrin repeat domain-containing protein 1) and *VLDLR* (very low-density lipoprotein receptor) genes (Fig. 5). Interestingly, a highly conserved region under positive selection on ECA10 (27.8–28.3Mb) also contained a cluster of Zinc finger protein-related genes, *ZNF135*, *ZNF274*, *ZNF606*, *C10H19orf18* (chromosome 10 C19orf18 homolog), and *ZNF671*.

SOS analysis of the breed group with the high risk of DSLD vs. the breed group with medium risk of DSLD identified a ∼1.9Mb region under positive selection on ECA18. This region contained the *COL5A2* (collagen type 5 alpha 2 chain) gene (Fig. 5). In this contrast, the ECA7 region also contained the *JUNB* (proto-oncogene, *AP-1* transcription factor subunit) and *PDRX2* (peroxiredoxin 2) genes.

Finally, our SOS results also showed that for comparison of the breed group with high risk of DSLD vs. the control breed group, there were ∼0.11, 0.52, and ∼1.1Mb regions with a high selection signal on the ECA2, 3, and 6, respectively, that did not contain genes whose function is known to be related to tendon biology. Evaluation of genomic regions under selection using analysis with the *F*_ST_ statistic identified regions shared with hapFLK analysis, particularly loci on ECA2, 10, and 23 (Fig. 5). *F*_ST_ results showed a more polygenic trend than hapFLK analysis.

### Association testing of breed risk groups using a liability ordinal threshold model

**Fig. 6.**
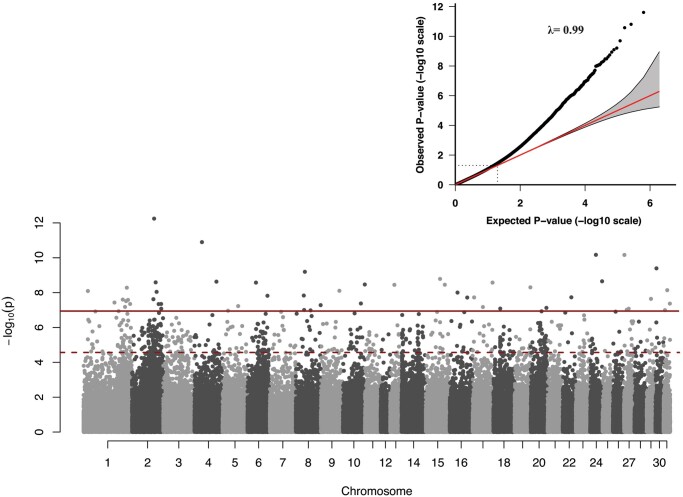
Manhattan plot of GWAS results from the liability estimates used as input for an ordinary linear mixed model of breed groups with differing risk of degenerative suspensory ligament desmitis. Each point represents a SNP. The first dashed dark red line represents the permuted threshold line and the second dark red solid line represents the genome-wide significance level for Bonferroni correction in −log10(*P*-value) scale. The top right plot shows the *Q*–*Q* plot of the genome-wide association, where the −log10-transformed observed *P*-values (*y*-axis) are plotted against –log10-transformed expected *P*-values (*x*-axis).

GWAS results showed many SNPs deviating from the null line, and 51 SNPs showed a −log10 *P*-value, larger than the Bonferroni threshold (Fig. 6). The estimated lambda value was ∼1 indicating that there is no systematic bias in the analysis. The QQ plot showed deviation of many observed *P*-values for significant SNPs from the null hypothesis, indicative of polygenicity in breed risk of DSLD.

The strongest single-variant associations between breed groups with differing risk of DSLD detected in the GWAS are reported in Supplementary File 2. We evaluated the 51 SNPs that passed the stringent Bonferroni correction threshold for associated genes by investigating 50kb up- and down-stream from each SNP; this analysis resulted in the identification of 46 genes (Table 3).

**Table 3.** Significant genome-wide SNPs identified using ordinal GWAS across four DSLD risk groups and genes located in the associated ±50kb window.

SNP position	*P*-Value	Chr.	Window (bp)	Genes
1:167,889,750	5.23E−09	1	167,839,750–167,939,750	None
1:16,979,620	8.12E−09	1	16,929,620–17,029,620	ATRNL1
1:152,659,086	2.48E−08	1	152,609,086–152,709,086	MEIS2
1:172,709,687	2.64E−08	1	172,659,687–172,759,687	NPAS3
1:165,399,279	3.08E−08	1	165,349,279–165,449,279	STXBP6
1:120,389,182	3.72E−08	1	120,339,182–120,439,182	**SEMA7A**
1:181,251,053	4.45E−08	1	181,201,053–181,301,053	None
1:161,411,374	6.58E−08	1	161,361,374–161,461,374	None
2:85,873,720	5.69E−13	2	85,823,720–85,923,720	None
2:92,260,610	2.58E−09	2	92,210,610–92,310,610	RAB33B/NAA15
2:95,726,382	9.07E−09	2	95,676,382–95,776,382	None
2:83,316,550	2.40E−08	2	83,266,550–83,366,550	LRBA/DCLK2
2:113,433,033	4.46E−08	2	113,383,033–113,483,033	CAMK2D/ANK2
2:103,773,160	4.54E−08	2	103,723,160–103,823,160	None
2:113,793,329	8.30E−08	2	113,743,329–113,843,329	ANK2
4:29,201,049	1.27E−11	4	29,151,049–29,251,049	None
4:85,780,520	2.34E−09	4	85,730,520–85,830,520	MKLN1
5:60,855,540	5.92E−08	5	60,805,540–60,905,540	None
5:21,231,893	1.09E−07	5	21,181,893–21,281,893	KCNK2
6:33,050,364	2.67E−09	6	33,000,364–33,100,364	CCND2
6:77,717,350	1.51E−08	6	77,667,350–77,767,350	SLC16A7
8:35,272,427	6.35E−10	8	35,222,427–35,322,427	LRRC30
8:31,344,118	1.47E−08	8	31,294,118–31,394,118	None
8:96,476,384	5.20E−08	8	96,426,384–96,526,384	ATP9B
8:32,610,990	9.96E−08	8	32,560,990–32,660,990	ZNF891/ZNF10
8:57,317,599	1.04E−07	8	57,267,599–57,367,599	CCDC178
9:72,122,737	7.83E−09	9	72,072,737–72,172,737	None
10:84,997,106	3.41E−09	10	84,947,106–85,047,106	NHSL1/CCDC28A/ECT2L
10:69,894,473	4.22E−08	10	69,844,473–69,944,473	TBC1D32
13:16,237,653	3.58E−09	13	16,187,653–16,287,653	AUTS2
15:55,512,469	1.63E−09	15	55,462,469–55,562,469	PPM1B
15:74,044,214	3.55E−09	15	73,994,214–74,094,214	None
16:30,408,620	9.94E−09	16	30,358,620–30,458,620	FHIT
16:68,091,834	1.91E−08	16	68,041,834–68,141,834	**COL6A5**
17:77,491,730	2.65E−09	17	77,441,730–77,541,730	**COL4A1**
17:6,212,612	1.88E−08	17	6,162,612–6,262,612	RNF6/CDK8
17:40,253,974	6.67E−08	17	40,203,974–40,303,974	None
18:27,056,892	8.22E−08	18	27,006,892–27,106,892	GTDC1
19:61,510,513	4.96E−09	19	61,460,513–61,560,513	None
20:61,064,890	7.51E−08	20	61,014,890–61,114,890	None
22:33,468,031	1.85E−08	22	33,418,031–33,518,031	PTPRT
24:22,791,028	6.75E−11	24	22,741,028–22,841,028	SPTLC2
24:46,690,329	2.23E−09	24	46,640,329–46,740,329	CEP170B/PLD4/AHNAK2/CLBA1
27:1,613,634	6.87E−11	27	1,563,634–1,663,634	PSD3
27:18,207,816	8.31E−08	27	18,157,816–18,257,816	SGCZ/MIR383
27:10,846,899	9.65E−08	27	10,796,899–10,896,899	None
29:17,669,169	2.26E−08	29	17,619,169–17,719,169	MALRD1
30:4,279,845	4.07E−10	30	4,229,845–4,329,845	**GREM2**/FMN2
31:14,453,924	7.19E−09	31	14,403,924–14,503,924	None
31:24,992,307	4.26E−08	31	24,942,307–25,042,307	None
31:6,076,336	1.01E−07	31	6,026,336–6,126,336	PACRG

Genes highlighted in bold underlined text may have biological influences on tendon and ligament homeostasis. Windows represent 50kb flanking regions around each associated SNP.

**Fig. 7.**
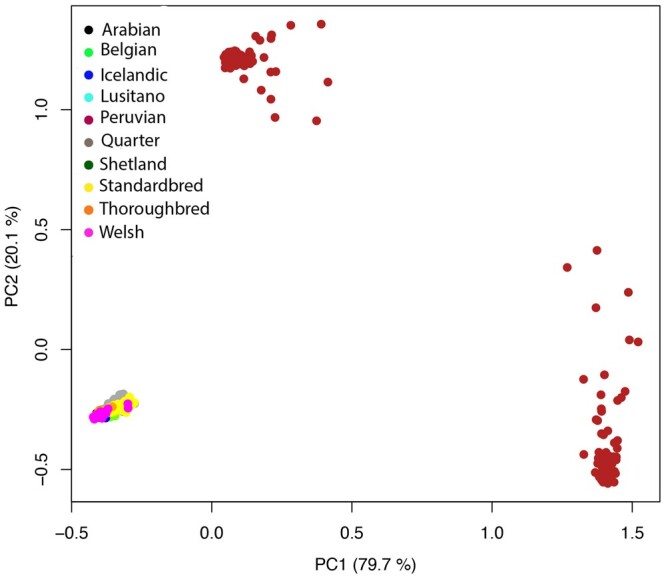
Principal component analysis (PCA) plot using the top 1,440 GWAS SNPs with *P*-values below the significance threshold determined by permutation testing for association with the breed risk of DSLD. In contrast to the single cluster of Peruvian Horses in Fig. 2, Peruvian Horses now separate into 2 clusters with the remaining breeds clustered together.

Several genes were identified with high relevance to tendon/ligament homeostasis (Table 3). Associated genes we found included *SEMA7A* (semaphorin 7A) on ECA1, *COL6A5* (collagen type VI alpha 5 chain) on ECA16, *COL4A1* (collagen type IV alpha 1 chain) gene on ECA17, and *GREM2* (gremlin 2 DAN family BMP antagonist) on ECA30. Finally, associations with zinc finger proteins were also identified on ECA8. When PCA was repeated using the 1,440 significant GWAS SNPs with *P*-values lower than the permutation testing threshold (*P *≤* *3.24E−5), we found that the first 2 PCs explained most of the genetic variation (Supplementary Fig. 7). The PH population separated into two clusters and the other horses in the remaining nine breeds cluster together (Fig. 7).

### Pathway analysis

Results of pathway analysis with Panther are presented in Supplementary File 3. Overrepresented functions for genes associated with differing breed risk of DSLD from SOS analysis included cellular and metabolic processes and binding. Highlighted pathways included signaling through the EGF receptor, inflammation mediated by chemokines and cytokines, nicotinic acetylcholine receptor, gonadotropin-releasing hormone receptor, and the wnt pathway. Overrepresented protein classes included nucleic acid metabolism proteins, metabolite interconversion enzymes, and gene-specific transcriptional regulators. Similar results were obtained from the GWAS analysis. Here, pathways enriched by GWAS-associated genes include integrin, inflammation mediated by chemokines and cytokines, PI3 kinase, ionotropic glutamate receptor, and cell cycle signaling.

## Discussion

### Categorical GWAS approach using breed-assigned risk levels

In a One Health comparative analysis undertaking research that is beneficial to both humans and animals, a genome-wide SOS analysis and a GWAS were used to identify genome-wide associations between breed groups categorized by differing risk of DSLD. Genes residing in candidate loci were evaluated for relevance to tendon/ligament injury. As a part of our SOS analysis, population structure within the data was evaluated. We confirmed homogeneity within horse breeds and heterogeneity between breeds in our sample population of 10 breeds based on PCA (Schaefer *et al.* 2017) and findings from the phylogenetic cladogram. LD decay within breeds generally decayed as previously described (Petersen *et al.* 2013; Schaefer *et al.* 2017). The admixture patterns in our results showed some admixture between the PH and other breeds with differing DSLD risk. We based risk categorization on clinical knowledge of the incidence of DSLD in different breeds (Young 1993; Mero and Scarlett 2005; Halper *et al.* 2006, 2011; Luo *et al.* 2016). We found that the PH, which was the only breed assigned a high risk of DSLD, formed a distinct cluster genetically based on PCA, but admixture analysis suggests sharing of ancestry with the SB, the AR, and the LU breeds. Here, it is important not to overinterpret admixture plots where recent genetic drift is strong (Lawson *et al.* 2018). The AR breed as a genetic root also formed a distinct cluster; results that align with knowledge of the origin of this horse breed (Cosgrove *et al.* 2020). This type of categorical GWAS research approach with breed-assigned risk levels has previously been used in dogs (Smith *et al.* 2019; Doherty *et al.* 2020), but not horses. Further validation of candidate genes is important as biologically relevant candidate genes may still represent false-positive discoveries (Pavlidis *et al.* 2012).

### Effective population size

The effectiveness of selection of a mutation depends on both the fitness effect of new beneficial mutations and the effective population size (Ne). We computed effective population size for each breed separately. The breeds we studied have small recent effective population size, except for the QH (Ne > 100). It has been suggested that a minimum Ne of 50–100 is needed for sustaining reproductive fitness in the short term (∼100 years) (Frankham *et al.* 2014). Horse breeds are largely defined by segregation of alleles because of founder effect, small effective population size, and maintenance of deleterious alleles through artificial selection (Gossmann *et al.* 2012).

### DSLD pathology

A key feature of DSLD is acellular accumulation of proteoglycans, such as aggrecan and decorin, replacing cells and collagen bundles, suggesting disturbance to matrix homeostasis (Halper *et al.* 2006, 2011; Schenkman *et al.* 2009; Young *et al.* 2018). Such features parallel tendon aging and tendinopathy in humans (Winnicki *et al.* 2020; Smith and McIlwraith 2021). A certain amount of proteoglycan turnover may be required to maintain normal tendon/ligament collagen assembly and matrix homeostasis by aggrecanases that are constitutively active in the tissue (Rees *et al.* 2009). In this regard, it is interesting to note that accumulation of *ADAMTS4* (∼chr5:32.4Mb) and *ADAMTS5* (∼chr26:25.1Mb) aggrecanases has been identified in DSLD-affected tendons suggesting sequestration of these enzymes (Plaas *et al.* 2011).

### Candidate genetic variants associated with differing breed risk of DSLD influence tissue aging

Variants in candidate genes discovered in the present study have the potential to influence SL homeostasis and matrix composition through effects on mechanotransduction, collagen assembly, and turnover and, consequently, contribute to DSLD pathology. *PIN1* (∼chr7:51.8Mb) is a gene with an important role in tendon aging in humans through modulation of diverse cellular functions; such effects may influence tendon degeneration (Chen *et al.* 2015). Aging is a dominant risk factor for tendon injury and impaired tendon healing, but the associated mechanisms are poorly understood. *PIN1* has regulatory effects on cellular aging; overexpression delays the progression of cellular senescence as indicated by the downregulation of senescence-associated β-galactosidase and enhanced telomerase activity (Chen *et al.* 2015; Lv *et al.* 2018). *PIN1* is also recognized to have a vital role in tendon stem/progenitor cell aging and its upstream miRNA is a prospective target for preventing progenitor cell aging (Dai *et al.* 2019). Mechanical loading influences expression of multiple miRNAs in tendon including downregulation of miRNA-140-5p (Mendias *et al.* 2012).

### Candidate genetic variants associated with differing breed risk of DSLD influence tissue mechanotransduction

Other genes associated with differing breed group risk of DSLD include *KANK1* and *KANK2*. The KANK protein family is characterized by an N-terminal KN motif, coiled-coil motifs, and 4–5 ankyrin repeat domains located in the C-terminus and are key regulators of adhesion dynamics (Kakinuma *et al.* 2008; Zhu *et al.* 2008). *KANK* is a novel Akt substrate and its interaction with 14–3–3 proteins is controlled by the phosphoinositide-3 kinase (PI3K)–Akt signaling pathway (Kakinuma *et al.* 2008); a pathway that is critically important to tendon mechanotransduction (Wang *et al.* 2018). *KANK* proteins act to decrease the RhoA-dependent formation of actin stress fibers and cell migration (Kakinuma *et al.* 2008; Seetharaman and Etienne-Manneville 2020). PI3K/Akt signaling regulates important cellular functions including apoptosis, cell growth, and cell migration; this pathway has an important role in tendon/ligament homeostasis and repair (Zhang *et al.* 2020). *KANK2*, located on ECA7, is a paralog of this gene and has similar physiological functions (Zhu *et al.* 2008; Gee *et al.* 2015) and likely similar effects on mechanotransduction. Disruption of *KANK1/2* function may have important novel effects on tendon/ligament mechanotransduction and collagen synthesis (Paradzik *et al.* 2020).

Other candidate tendinopathy genes associated with differing breed group risk of DSLD identified in this study include *JUNB*, and *SEMA7A.* The transcription factor *JUNB* has been linked to type I collagen disruption in fibrous connective tissue disease (Ponticos *et al.* 2015) and involvement in signaling pathways for tendon (Tan *et al.* 2020) and muscle (Verbrugge *et al.* 2018) homeostasis. Semaphorins are cell surface signaling molecules that regulate cell migration. *SEMA7A* may influence mechanotransduction in tendon/ligament (Spencer and Lallier 2009), but more studies are needed. *SEMA7A* also has a role in TGF-beta1-mediated tissue remodeling and fibrosis (Kang *et al.* 2007).

### Candidate genetic variants associated with differing breed risk of DSLD influence matrix composition

Interestingly, we also identified additional genes associated with differing breed group risk of DSLD that influence low abundance fibrillar collagen homeostasis in tendon/ligament. Fibrillar collagen molecules are trimers that can be composed of 1 or more types of alpha chains. SNP associations within the *COL5A2* gene on ECA18 and *COL5A3* gene on ECA7 were identified from our SOS analysis. Type V collagen is found in tissues containing type I collagen and acts to regulate assembly of heterotypic fibers composed of both type I and type V collagen (Mak *et al.* 2016). In humans, Ehlers–Danlos syndrome associated with connective tissue hyperelasticity and fragility is linked with heterozygous mutations in *COL5A2* (Chiarelli *et al.* 2019); *COL5A3* mutations have also been implicated in the syndrome (Imamura *et al.* 2000). Collagen V plays an important role in regulating fibrillogenesis and associated recovery of mechanical integrity in tendons after injury (Johnston *et al.* 2017). A proteomic composition analysis of the muscle, tendon, and junction tissues showed that *COL5A3* as a potential marker for the muscle-tendon junction, a highly interdigitated interface that seamlessly transfers muscle-generated forces to the tendon (Jacobson *et al.* 2020). These observations suggest more detailed study of myofibers in the equine SL may be indicated. Risk of Achilles tendinopathy is influenced by interactions between extracellular matrix proteins and cell signaling pathways and this risk is influenced by *COL5A2* and *COL5A3* variants (Bancelin *et al.* 2015; Bittermann *et al.* 2018; Jacobson *et al.* 2020). The proportion of collagen type V is increased in Warmblood injured SL, a breed registry that is predisposed to DSLD (Shikh-Alsook *et al.* 2015).

We also found that SNPs within *COL4A1* and *COL6A5* on ECA16 and 17 were associated with breed group differences in the risk of DSLD. Collagen IV is a major component of basement membrane. The α1(IV) chain, encoded by *COL4A1*, is expressed ubiquitously and associates with the α2(IV) chain to form the α1α1α2(IV) heterotrimer. Analysis of skeletal muscle from *COL4A1* mutant animals identified a muscular dystrophy phenotype with myofiber atrophy, centronucleation, focal inflammatory infiltrates, and fibrosis (Guiraud *et al.* 2017). Collagen VI is an extracellular matrix protein with critical roles in maintaining muscle and skin integrity (Lamande and Bateman 2018). The role of *COL6A5* mutations in the development of myopathy and fibrosis is not understood (Sabatelli *et al.* 2011), but the expression of *COL6A5* is decreased in the skin of DSLD-affected horses (Haythorn *et al.* 2020).

### Candidate genetic variants associated with differing breed risk of DSLD influence lipid metabolism

There are few studies specifically focused on the role of low-density lipoprotein (LDL) and very low-density lipoprotein (VLDL) mutations in tendon/ligament homeostasis, but there is strong evidence that links these 2 lipoproteins with tendinopathy in familial hypercholesterolemia (Tilley *et al.* 2015; Aljenedil *et al.* 2018). Elevated plasma cholesterol, low-density lipoprotein cholesterol and triglyceride, and lower high-density lipoprotein cholesterol are associated with an increased risk of tendinopathy and xanthoma formation because of *LDLR* mutations (Raal and Santos 2012; Tilley *et al.* 2015). Linkage of *VLDLR* variants with tendinopathy has not been established.

### Other candidate genetic variant effects on differing breed risk of DSLD

Other candidate tendinopathy genes associated with breed group differences in DSLD risk identified in this study include *PRDX2* and *GREM2*. Upregulation of the antioxidant enzyme *PRDX2* has a functional role in oxidative stress (Chu *et al.* 2013; Jeong *et al.* 2018) and has been linked to development of rotator cuff tendinopathy in humans (Thankam and Agrawal 2021). *GREM2* is a *BMP* antagonist that is regulated by the circadian clock; *GREM2* has been linked to the development of tendon calcification and the development of tendinopathy (Yeung *et al.* 2014).

Many associations were identified with zinc finger proteins. Zinc finger proteins are among the most abundant proteins in eukaryotic genomes and their functions are extraordinarily diverse (Laity *et al.* 2001; Gurgul *et al.* 2019), but a role in tendinopathy has not been clearly defined.

In general, candidate genes associated with breed group differences in DSLD risk were supported by pathway analysis results. Pathway analysis suggested that disturbances to mechanotransduction are an important component of DSLD pathogenesis. It is also interesting to note that structural and extracellular matrix proteins were not highlighted in our pathway analysis.

### Study limitations regarding use of breed repository SNP data

More work validating our research findings and expansion of the breed repository SNP set is needed to fully understand the usefulness of this approach, since biologically relevant candidate genes may still represent false-positive discoveries (Pavlidis *et al.* 2012). Our current data set is limited to a single breed with high risk of DSLD, the PH, which showed some admixture with other breeds with differing DSLD risk, such as the AR, the SB, and the LU. The extent to which contrasts with other breeds may reflect variant differences between breed characteristics as opposed to causal associations with DSLD is unclear and needs more investigation. However, associations with candidate tendinopathy genes are biologically plausible. When PCA using the top GWAS SNPs was repeated, the PH population segregated into 2 clusters likely with differing within-breed risk of DSLD. This observation supports the categorical GWAS approach we have used in this study. These findings would be strengthened by further across breed analysis after addition of other high-risk breeds to the data set. Enlargement of the breed repository data set over time could be very valuable with regarding to analysis of other disease phenotypes in horses where clinical knowledge includes information on breed incidence or breed categorical risk of disease. Additional analysis through case–control association within the PH breed using individually phenotyped horses would also provide further validation for specific candidate genes.

## Conclusions

Use of categorical GWAS with breed-assigned risk levels is a potentially useful research approach in horses. Our findings contribute to knowledge of the genetic background that explains differing breed risk of DSLD. Using a One Health comparative analysis investigating SOS and categorical GWAS of breeds of horse with different breed risk of DSLD, our results suggest that DSLD is a complex disease with a polygenic architecture. We have identified several novel candidate genes associated with elevated breed risk of DSLD that are also novel candidates for human tendinopathy. When taken together with existing knowledge of the pathology of the disease, our findings suggest that DSLD pathogenesis is associated with disturbances to SL homeostasis where matrix responses to mechanical loading are perturbed through disturbances to aging in tendon (*PIN1*), mechanotransduction (*KANK1*, *KANK2*, *JUNB*, *SEMA7A*), collagen synthesis (*COL4A1*, *COL5A2*, *COL5A3*, *COL6A5*), matrix response to hypoxia (*PRDX2*), and lipid metabolism (*LDLR*, *VLDLR*). Our results do not suggest that proteoglycan turnover in SL matrix is a primary factor in the disease pathogenesis. Candidate loci can now be specifically characterized using whole-genome sequencing for mutation discovery in both humans and the equine DSLD spontaneous tendinopathy model. These results are also encouraging regarding future development of a genetic risk test or prediction model to predict DSLD risk in horses before onset of clinical signs. To avoid inadvertent use of affected individuals for breeding, genetic testing will need a polygenic risk scoring approach.

## Supplementary Material

jkac179_Supplementary_Data

## Data Availability

Genotype and phenotype data are available at figshare: https://doi.org/10.25387/g3.20240223. Genotype and phenotype data are presented in a PLINK binary (.bed, .bim, .fam) format. The bed files contain genotyping information in binary format. The bim files contain SNP information. The fam files contain phenotypic information for each horse breed. Supplementary materials are also available at FigShare. Supplementary File 1 contains supplementary methods and results. Supplementary File 2 contains significant SNP GWAS association results. Supplementary File 3 contains significant pathway analysis results.
