# Supplementary material for: Selection signature analyses and genome‐wide association reveal genomic hotspot regions that reflect differences between breeds of horse with contrasting risk of degenerative suspensory ligament desmitis
Source: G3 (Bethesda). 2022 Jul 22;12(10):jkac179. doi: 10.1093/g3journal/jkac179 (PMC9526059; doi:10.1093/g3journal/jkac179)
Supplement: jkac179_Supplementary_Data [file jkac179_supplementary_data.docx]

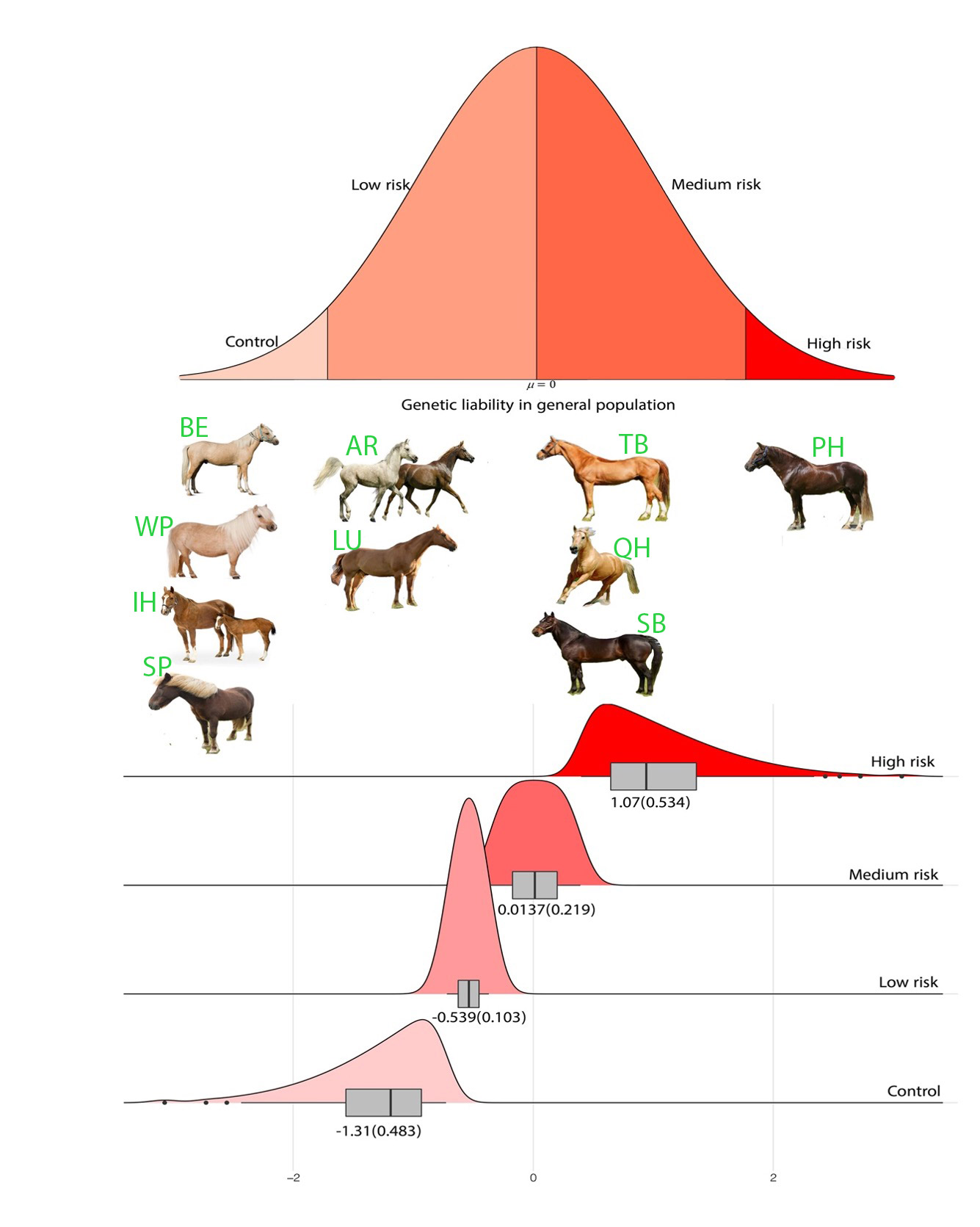


**Figure S1.** The hypothetical genetic distribution of liabilities across genetic risk groups. Control included the Belgian (BE), Icelandic Horse (IH), Shetland Pony (SP) and Welsh Pony (WP) breeds; low risk included the Lusitano (LU) and Arabian (AR) breeds; medium risk included the Standardbred (SB), Thoroughbred (TB), Quarter Horse (QH) breeds, and high risk consisted of the Peruvian Horse (PH). The bottom plot showed distributions of liabilities for each risk group separately estimated by a Bayesian linear mixed model. The numbers are mean (standard deviation) for each distribution.

**
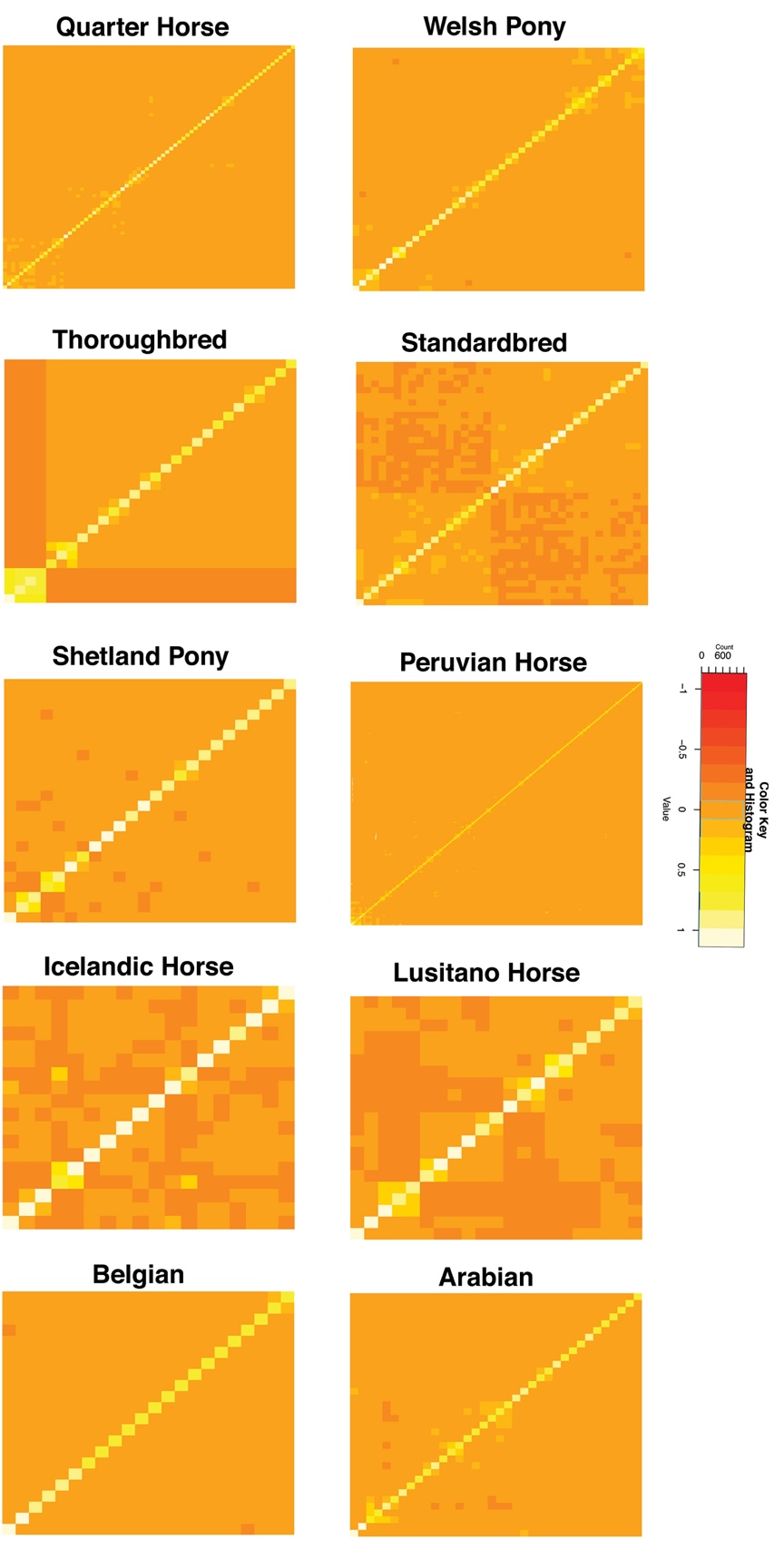
**

**Figure S2**. Genomic relationship matrix heatmap plotted for each breed of horse in the study. The colors represent the strength of the relationship between individual horses within a breed.

**Figure S3**. The plot shows the variance explained by the first ten principal components. The solid line represents the variance reduction trend over all ten components. The analysis used all SNPs included in the GWAS.


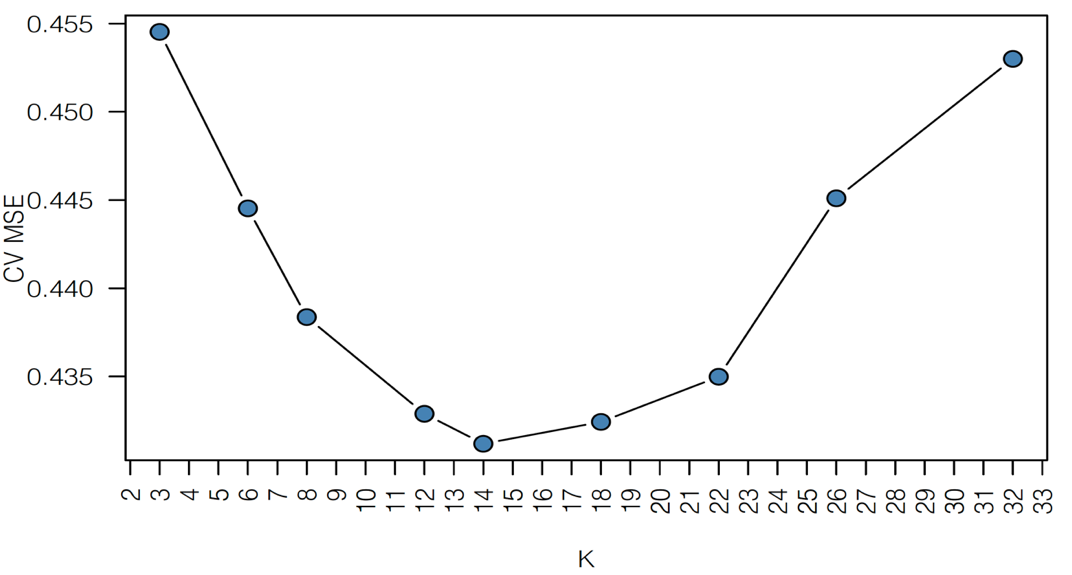


**
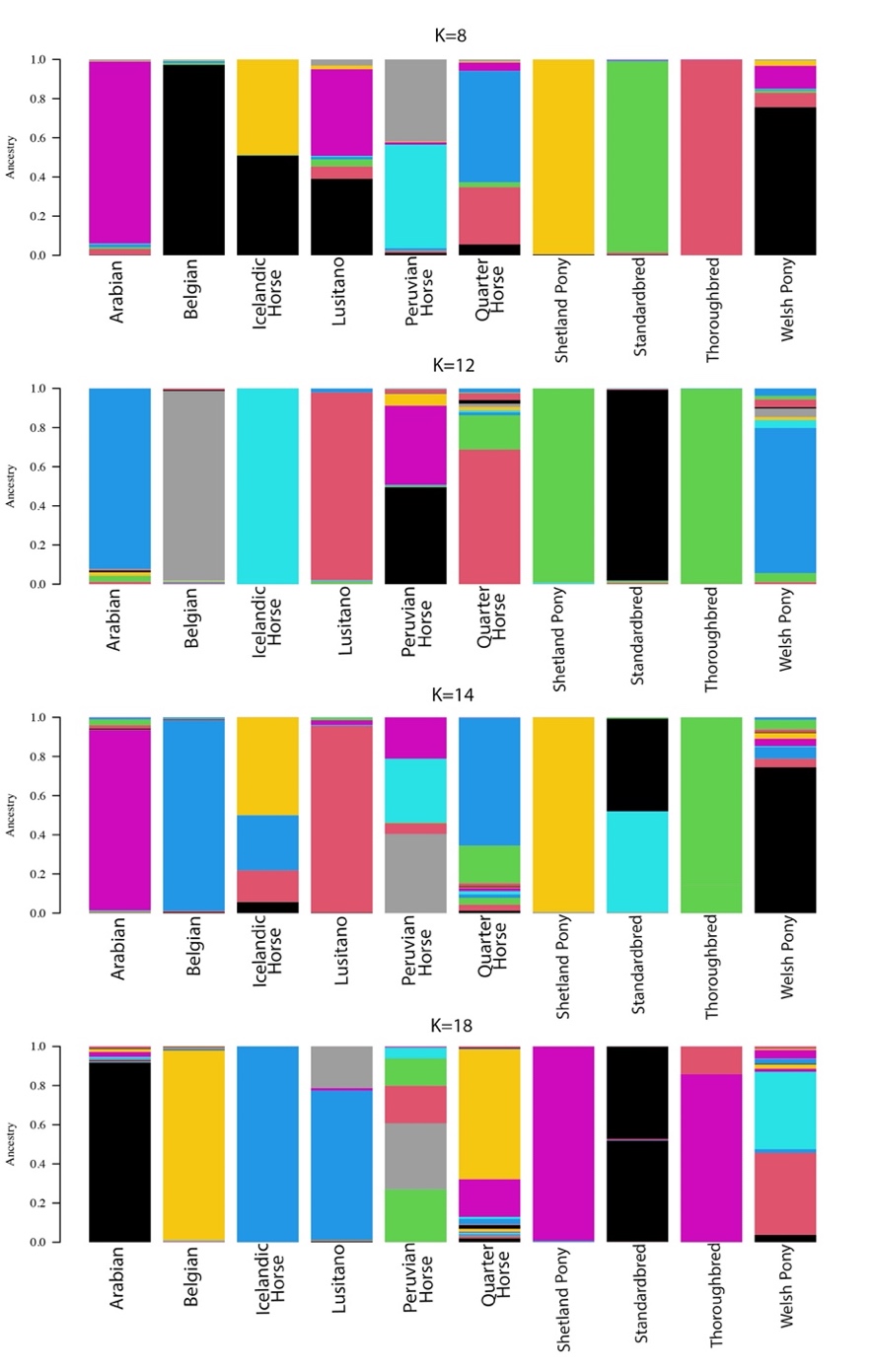
**

**Figure S4**. Admixture output for the four lowest mean squared error (MSE) values obtained by cross validation over K = 8, 12, 14, and 18. The lowest value was at K=14. Ancestry admixture proportion within each breed is represented by different colors. In all these scenarios, the Standardbred, Peruvian Horse, Lusitano, and Arabian showed some degree of ancestral gene flow based on admixture analysis.


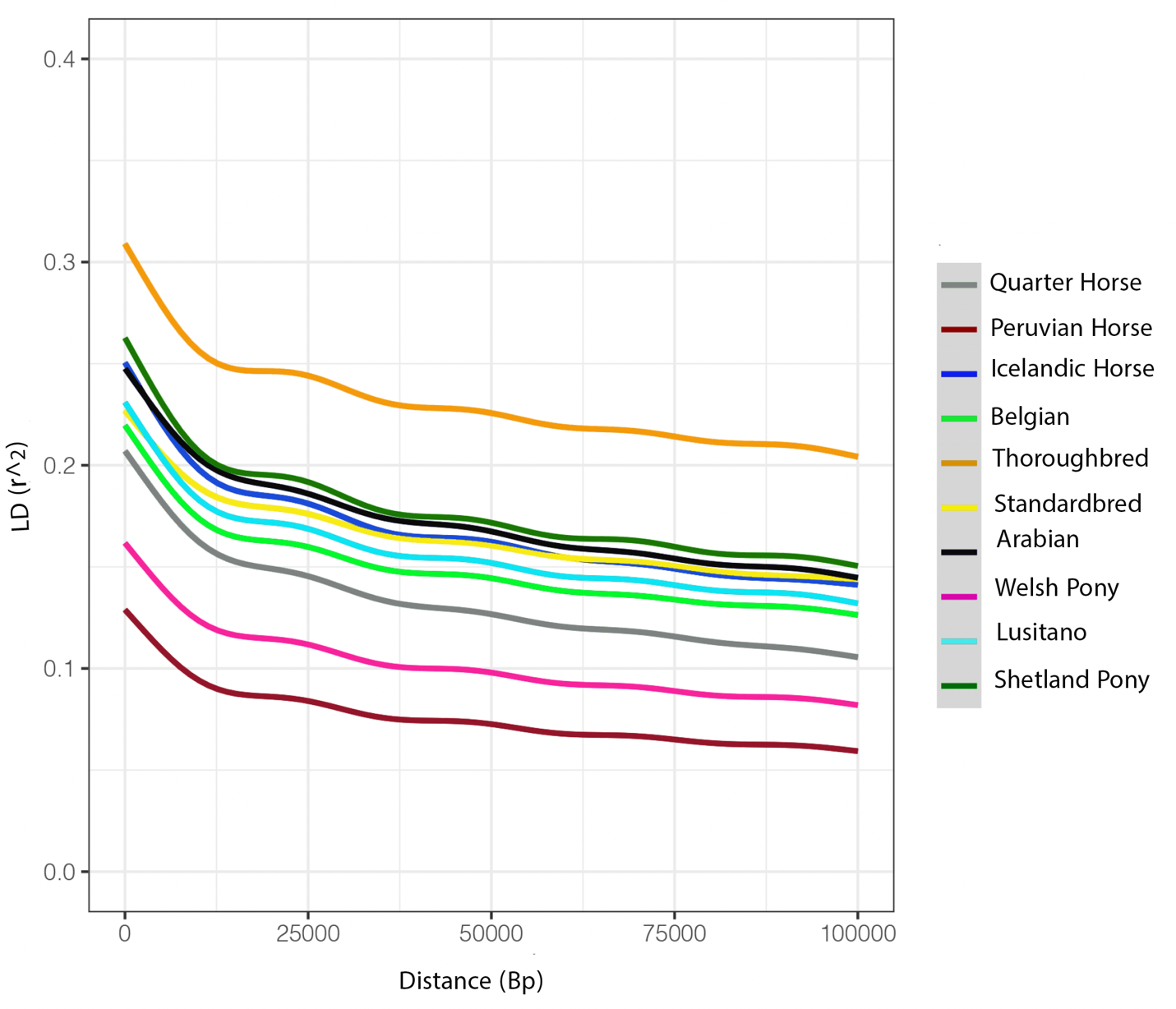


**Figure S5**. The decay of LD by distance in ten horse breeds plotted for marker distances up to 100 kb. The y-axis shows distribution of average pairwise r^2^ values.


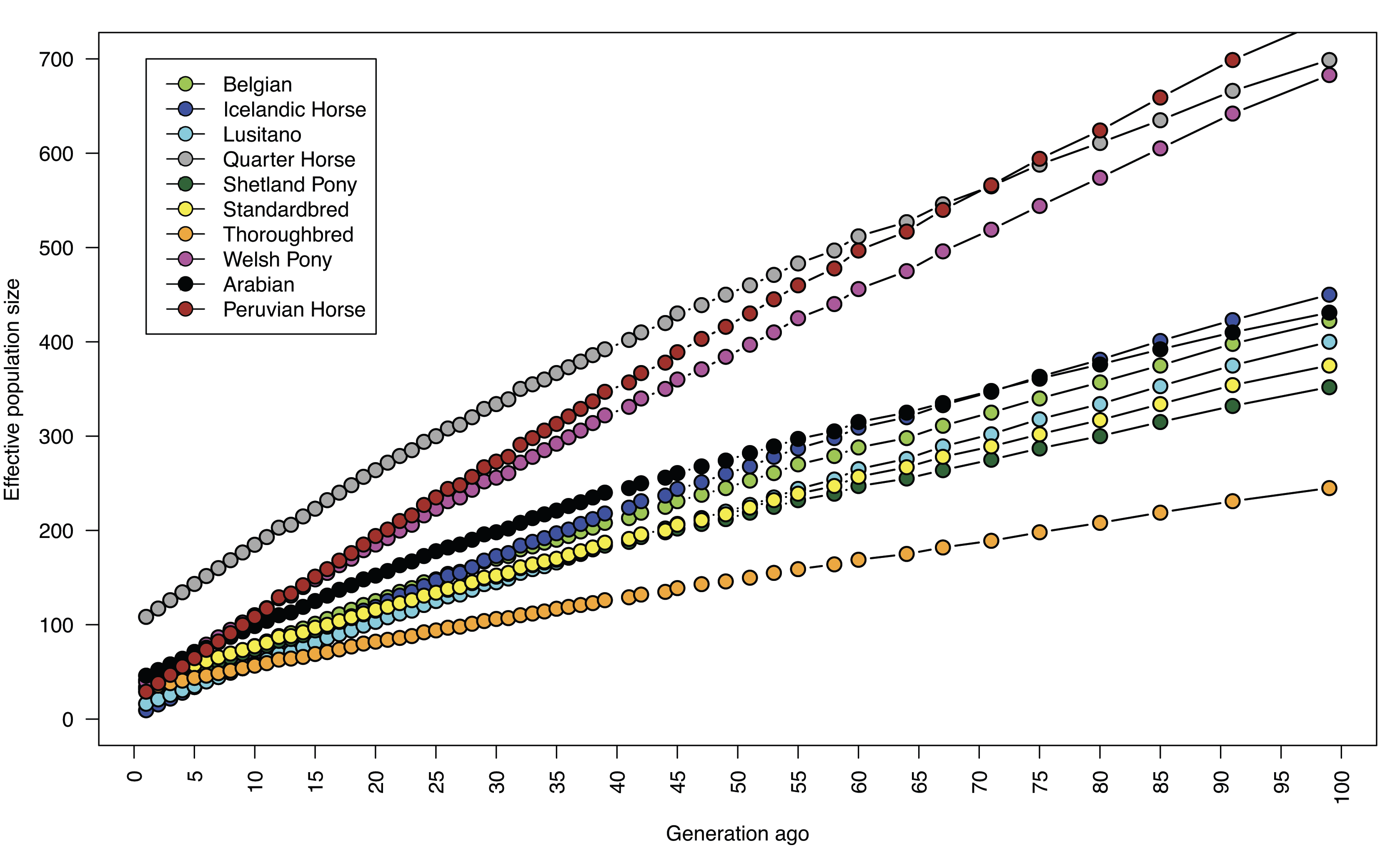


**Figure S6**. Estimated effective population sizes (Ne) in ten horse populations over 100 generation.


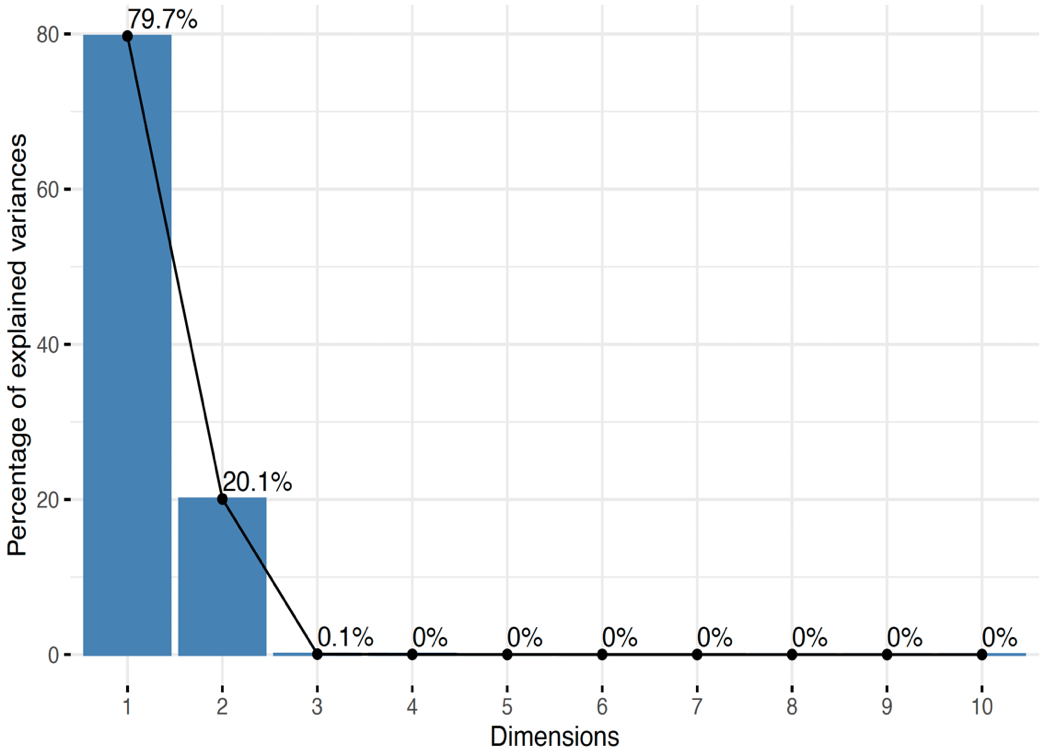


**Figure S7**. The plot shows the variance explained by the first ten principal components. The solid line represents the variance reduction trend over all ten components. The analysis used only the DSLD-associated significant GWAS SNPs.
